# Will Technology and Artificial Intelligence Make the Primary Care Doctor Obsolete? Remember the Luddites

**DOI:** 10.3389/fmed.2022.878281

**Published:** 2022-04-26

**Authors:** Arch G. Mainous

**Affiliations:** ^1^Department of Health Services Research, Management and Policy, University of Florida, Gainesville, FL, United States; ^2^Department of Community Health and Family Medicine, University of Florida, Gainesville, FL, United States

**Keywords:** primary care, artificial intelligence, workforce, health services research, nursing

Although primary care is valued by society, increasing the primary care physician workforce has begun to be less of a focus of both policymakers and healthcare systems. In particular, new virtual technology and artificial intelligence combined with an interest in increasing a broader group of professions including nurse practitioners, pharmacists, physician assistants, behavioral health specialists, and dental professionals to deliver primary care may render the primary care physician obsolete (1). New technology will play a large role in providing primary care with these teams. Moreover, many scientists working in artificial intelligence believe that core functions of medical professionals such as patient monitoring, diagnosis and prognosis will be transformed by technology and algorithms for care (2, 3). It has been argued that new technology, virtual visits and artificial intelligence will transform health care and can help the primary care physician better deliver services (4–6). An unintended consequence of new technology and artificial intelligence combined with a broader primary care workforce may unfortunately be a decrease in the number of primary care physicians rather than just an aid to help primary care physicians do better.

Perhaps the new technology will increase access to care but will it actually lead to a replacement of the primary care physician by technology and other health professionals? There is some disagreement among stakeholders as to whether primary care physicians will become obsolete due to artificial intelligence and new technology (7, 8). The results of those surveys and Delphi groups are likely incomplete in their conceptualization of the implications of the new technology for primary care physicians because in discussing this issue they don't consider the role of insurers and corporations in providing healthcare and deciding on the needed workforce.

An argument against technology and artificial intelligence as a threat to the primary care physician workforce may strike one as a statement of a Luddite. That would be correct but not based on the common definition of a Luddite. A Luddite is commonly thought of as one who is opposed to new technology. However, the history of the Luddites is a bit different and nuanced and very pertinent to this discussion. Luddites feared that new technology and more importantly, the way that technology would be used by the factory owners was exceptionally important. They were concerned that factory owners, in an effort to increase profits, would implement strategies with the new technology to replace workers' roles in their industry (9). It wasn't the invention of the new machines that agitated the Luddites and moved them to action. It was how the factory owners began using these new machines to displace and disempower workers (10).

The Luddites were against manufacturers who used machines in what they called “a fraudulent and deceitful manner” to get around standard labor practices (11). The Luddites wanted to create high-quality goods but they wanted these machines to be run by workers who had gone through an apprenticeship and got paid decent wages.

It is important to discuss several new models of delivering primary care to see where new technology, artificial intelligence and workforce preferences by employers, particularly leading to decreased labor costs may lead to the obsolescence of the primary care physician. Virtual-first primary care and patient self-administered histories are useful examples to present which dovetail with the experience of the Luddites.

## Virtual-First Primary Care

Virtual-first means that patients will use telehealth as the first option for primary care services. This is a rapidly increasing strategy of delivering primary care that is becoming particularly popular with insurers and patients. Perhaps it isn't specifically stated as the primary motivation, but definitely an underlying rationale for the growing phenomenon of “virtual-first” primary care is to cut costs (11, 12). Health plans and insurers are interested because the cost savings are substantial and the virtual-first plans are priced up to 20% lower than other traditional plans offered by the same insurer (12).

The encounter can be provided by a primary care physician or other primary care professional. Evidence suggests that primary care provided by nurse practitioners costs less than primary care provided by physicians (13). It would follow that if an underlying motivation for virtual-first primary care is to decrease costs then decreasing labor costs by using nurse practitioners rather than physicians to deliver primary care would be consistent with this strategy. Consequently, the use of primary care physicians to deliver virtual-first primary care would likely be less attractive than non-physician providers to the corporation in this new healthcare delivery system.

## Online Visits With Only a Presenting Complaint/Medical History

An additional feature of the new virtual-first and artificial intelligence healthcare delivery system is the extensive use of decision making based on a presenting complaint and medical history without a physical exam. Virtual visits for primary care are structured primarily with a patient talking to a provider without a physical exam or biomarker data. This strategy still requires a human provider, and their affiliated labor costs, to elicit the presenting complaint and take the history. Some health systems may use electronic portals where patients answer questions with branching logic to move toward a differential diagnosis or need for additional biomarkers. The creation of the algorithm with the corresponding accompanying questions for the patient is based on input from experts and pattern recognition. This information can be collected through an electronic interface without the need to pay a provider to collect the medical history face-to-face. This would decrease the labor costs for collecting the data.

For example, both Rory (https://www.hellorory.com/) for women's health and Roman (https://www.getroman.com/) for men's health provide prescription medication through online visit portals. They begin with a modified medical history collected through a series of self-report questions and branching logic. These modules have different questions based on the chosen ailment (e.g., allergies, erectile dysfunction, inadequate eyelashes). The patient can do everything totally through the interface without a video interaction with a healthcare provider. A video chat with a healthcare provider happens only in states with laws requiring it. In this structure, healthcare labor costs, and the need to employ primary care physicians is clearly minimized.

## Where Do We Go From Here?

This brings us back to the Luddites. If the decisions for modifying the healthcare system around new technology is driven by the desire of insurers and health systems to decrease costs, particularly labor costs, then the resulting system will likely need fewer and fewer primary care physicians. The Luddites wanted to produce high-quality goods but they also wanted higher trained workers to be used, valued and paid accordingly.

It is unclear whether the use of virtual-first primary care without a physical exam provided by nurse practitioners or other health professionals, or online visits without human interaction will lead to better or worse health outcomes. That is an open question and a needed focus of research. Nevertheless, this technology driven change in the delivery system will likely lead to fewer primary care physicians. Oh, and what happened to the Luddites? The factory owners prevailed.

## Author Contributions

The author confirms being the sole contributor of this work and has approved it for publication.

## Conflict of Interest

The author declares that the research was conducted in the absence of any commercial or financial relationships that could be construed as a potential conflict of interest.

## Publisher's Note

All claims expressed in this article are solely those of the authors and do not necessarily represent those of their affiliated organizations, or those of the publisher, the editors and the reviewers. Any product that may be evaluated in this article, or claim that may be made by its manufacturer, is not guaranteed or endorsed by the publisher.

## References

[1] National National Academies of Sciences Engineering and Medicine. Implementing High-Quality Primary Care: Rebuilding the Foundation of Health Care. Washington, DC: The National Academies Press (2021). 34251766

[2] Hinton G. Deep learning-a technology with the potential to transform health care. J Am Med Assoc. (2018) 320:1101–2. 10.1001/jama.2018.11100 30178065

[3] Yu KH Beam AL Kohane IS. Artificial intelligence in healthcare. Nat Biomed Eng. (2018) 2:719. 10.1038/s41551-018-0305-z 31015651

[4] Liaw W Kakadiaris IA Yang Z. Is artificial intelligence the key to reclaiming relationships in primary care? Am Fam Physician. (2021) 104:558–9. 34913641

[5] Liaw W Kakadiaris IA. Primary care artificial intelligence: a branch hiding in plain sight. Ann Fam Med. (2020) 18:194–5. 10.1370/afm.2533 32393552 PMC7214005

[6] Ford E Edelman N Somers L Shrewsbury D Lopez Levy M van Marwijk H. Barriers and facilitators to the adoption of electronic clinical decision support systems: a qualitative interview study with UK general practitioners. BMC Med Inform Decis Mak. (2021) 21:193. 10.1186/s12911-021-01557-z 34154580 PMC8215812

[7] Blease C Kaptchuk TJ Bernstein MH Mandl KD Halamka JD DesRoches CM. Artificial intelligence and the future of primary care: exploratory qualitative study of UK general practitioners' views. J Med Internet Res. (2019) 21:e12802. 10.2196/12802 30892270 PMC6446158

[8] Blease C Kharko A Locher C DesRoches CM Mandl KD. US Primary care in 2029: a Delphi survey on the impact of machine learning. PLoS ONE. (2020) 15:e0239947. 10.1371/journal.pone.0239947 33031411 PMC7544100

[9] Conniff R,. What the Luddites Really Fought Against. Smithsonian Magazine. (2011). Available online at: https://www.smithsonianmag.com/history/what-the-luddites-really-fought-against-264412/ (accessed February 22, 2022).

[10] Randall A. The 'lessons' of Luddism. Endeavor. (1998) 22:152–5. 10.1016/S0160-9327(98)01145-4

[11] Whitehead DC Mehrotra A. The growing phenomenon of “virtual-first” primary care. JAMA. (2021) 326:2365–6. 10.1001/jama.2021.21169 34807253

[12] Connolly C Hwang C. Nonprofit health plans launch telehealth-first options to increase access and affordability. NEJM Catal Innov Care Deliv. (2021). 10.1056/CAT.21.0031

[13] Razavi M O'Reilly-Jacob M Perloff J Buerhaus P. Drivers of cost differences between nurse practitioner and physician attributed medicare beneficiaries. Med Care. (2021) 59:177–84. 10.1097/MLR.0000000000001477 33273295 PMC7899223

